# On the Efficacy of a CBT-I-Based Online Program for Sleep Problems: A Randomized Controlled Trial

**DOI:** 10.3390/clockssleep5040039

**Published:** 2023-10-09

**Authors:** Esther-Sevil Eigl, Theresa Hauser, Pavlos I. Topalidis, Manuel Schabus

**Affiliations:** 1 Laboratory for Sleep, Cognition & Consciousness Research, Department of Psychology, Paris-Lodron University of Salzburg, 5020 Salzburg, Austria; esther-sevil.eigl@plus.ac.at (E.-S.E.);; 2 Centre for Cognitive Neuroscience Salzburg (CCNS), Paris-Lodron University of Salzburg, 5020 Salzburg, Austria

**Keywords:** insomnia, CBT-I, internet based, sleep problems, digital treatment, randomized controlled trial, polysomnography, sleep intervention, online program, sleep improvement

## Abstract

There is an urgent need for easily accessible treatment options for sleep problems to reduce the current treatment gap in receiving cognitive behavioral therapy for insomnia (CBT-I). Using a randomized controlled trial, we evaluated the efficacy of a CBT-I-based online program on sleep. Fifty-three volunteers (21–71 years; *M_Age_* = 44.6 ± 12.5; 27 female) suffering from impaired sleep were randomly allocated either to the experimental group (EG, *n* = 27) or to an active control group (CG, *n* = 26). The EG participated in a 6-week CBT-I-based online program, while the CG received psychoeducation and sleep hygiene instructions. Sleep was assessed both objectively via ambulatory polysomnography (PSG) as well as subjectively via questionnaires at three time points (baseline, pre- and post-intervention). A one-month follow-up assessment was performed using questionnaires. The EG showed small but reliable improvements from pre- to post-intervention in PSG-derived wake after sleep onset (from 58.6 min to 42.5 min; *p* < 0.05) and sleep efficiency (from 86.0% to 89.2%; *p* < 0.05). Furthermore, subjective sleep quality (assessed via Pittsburgh Sleep Quality Index) improved significantly during intervention (*p* = 0.011) and follow-up (*p* = 0.015) in the EG alone. The Insomnia Severity Index decreased from pre- to post-intervention in both groups (EG: *p* = 0.003, CG: *p* = 0.008), while it further improved during follow-up (*p* = 0.035) in the EG alone. We show that a CBT-I-based online program can improve sleep not only subjectively but also objectively and can be a viable alternative when face-to-face interventions are not available.

## 1. Introduction

When restorative sleep no longer occurs naturally but is frequently impaired, sleep can no longer fulfil its functions sufficiently. Sleep disorders have become one of the most common health complaints in society [1,2,3,4]. While 30% of the general population suffer from sleep problems occasionally without meeting the criteria for insomnia disorder [5], up to 10% of the European adult population suffer from symptoms of chronic insomnia, including difficulties of falling asleep and/or maintaining sleep, early morning awakenings and severe daytime impairments [6,7,8]. This can have a significant impact on a person’s subjective well-being, quality of life [9] and global functioning [10] and is related to numerous negative consequences as well as health concerns such as increased risks of cardiovascular diseases [11], diabetes [12], mental disorders like anxiety [13,14] and depression [15] as well as increased substance abuse [16] and suicidal tendencies [17]. The immune system, cognitive functioning and memory are furthermore just a few of the physiological and neurobehavioral processes that can be significantly impacted by insufficient sleep [18,19,20]. Besides the individual burden, insomnia is associated with substantial costs for the health care system [21], which range between EUR 40 and 50 billion annually in Germany alone [22].

According to European and American guidelines [23,24], the recommended first-line treatment for adults suffering from chronic insomnia is the cognitive behavioral therapy for insomnia (CBT-I). This psychotherapeutic treatment consists of the following main components: psychoeducation, relaxation techniques, methods of sleep–wake structuring (e.g., stimulus control, sleep restriction) as well as cognitive techniques to change dysfunctional beliefs and to reduce nocturnal rumination [24,25]. Traditionally, it is delivered in six to eight weekly face-to-face group sessions. In long-term follow-up assessments, onsite CBT-I has been shown to be effective in improving subjective sleep parameters for up to 12 months [25,26] and is therefore considered to be more effective in the long run than sleep medication [27,28,29]. Due to a lack of trained providers, however, applying and disseminating CBT-I is a major challenge [30]. Indeed data from industrialized countries suggest that only about 10% of those being diagnosed receive the recommended first-line treatment [31], while up to 70% of those affected never even discuss the problem with a health care professional [32,33].

Lately, efforts have been made to find solutions to overcome the limited accessibility of CBT-I. In this context, scalable digital therapies and interventions have gained increasing influence in recent years. These have the advantage of being cost-effective and available at any time. Furthermore, the offer is overall low-threshold, which means that it does not require a visit to the doctor or therapist [34,35]. Using a fully automated web-based program guided by a virtual avatar, improvements were found in the subjective sleep parameters sleep onset latency (SOL), wake after sleep onset (WASO), sleep efficiency (SE) as well as in self-reported daytime functioning when compared with both placebo and treatment as usual [36]. Similarly, another fully automated web-based program called SHUTi led to improvements in insomnia severity, SOL and WASO [37]. The effects were found to be stable in a one-year follow-up assessment. Apart from improvements in insomnia severity, ameliorations in daytime fatigue, psychological distress and dysfunctional beliefs about sleep have also been ascertained in an large-scale investigation [38] using an unguided web-based program. A meta-analysis including eleven randomized controlled trials confirmed the robustness of the effects of internet-delivered CBT-I (iCBT-I), reporting a large global effect size (*g* = 1.09, CI 0.74–1.45) for insomnia severity and various sleep-related parameters [30]. This finding was underpinned by further taking into account long-term efficacy [39]. Investigating the efficacy of different CBT-I settings, a recent meta-analysis yielded large effect sizes regarding insomnia severity for individual onsite CBT-I, group-delivered face-to-face (F2F) CBT-I, telehealth and guided bibliotherapy as well as medium effect sizes regarding both guided and unguided iCBT-I (CBT-I provided via a website/web application or an internet browser) [40]. Synchronous settings (i.e., individual onsite, group-delivered, telehealth), however, resulted in the largest effect sizes.

Although there is strong empirical support for the effects of CBT-I programs on the subjective parameters of sleep, most studies lack the integration of objective sleep data. The investigation of objectively assessed data is, however, important, as the examination of objective data shows that most insomniacs exhibit disturbed sleep physiology, continuity as well as altered sleep architecture when compared with healthy controls [41]. Therefore, only the additional investigation of objectively derived data (i) can reveal if an intervention manages to improve sleep objectively and (ii) is thus able to ease the associated negative consequences of insufficient sleep on a physiological level (immune system, glymphatic system, etc.).

Although literature dealing with the effects of sleep interventions on objective sleep parameters is sparse, a few studies concentrating on insomnia sufferers have reported objective sleep benefits after the application of CBT-I. A clinical review focusing on the effects of (F2F) CBT-I in comorbid insomnia found small effects regarding an amelioration of WASO and SOL when evaluating studies which partly used actigraphy as a more economical way of obtaining objective sleep data [42]. By collecting ambulatory polysomnography (PSG) data, one study reported improvements in WASO and SE as well as a more rapid decline in EEG delta power in the course of the night while comparing a 6-week CBT-I group with a placebo control group [43]. Similarly, various objective sleep parameters (TST, SE, WASO) were found to be improved with large to medium effect sizes after participation in an 8-week F2F CBT-I program delivered in individual weekly 60 min sessions [44]. While additionally concentrating on changes in sleep architecture and EEG power densities, reduced beta and sigma activity and increased Slow Wave Activity as well as REM- and Slow Wave Sleep durations were discovered after CBT-I as compared to before. The authors concluded that CBT-I may improve homeostatic sleep regulation by enhancing slow wave activity, may intensify sleep pressure and may positively act upon central nervous system (CNS) hyperarousal, which is known to be elevated in insomnia sufferers [45,46]. Finally, a recent meta-analysis (only including RCTs) systematically comparing several CBT-I settings reports intervention effects on objective SOL for F2F CBT-I, telehealth and sleep hygiene education [40]. For SE and WASO, no effects were observed, which may, however, have been caused by the non-inclusion of the previously mentioned studies due to the exclusion criteria applied.

Apart from causing changes in sleep parameters and architecture, CBT-I can help to reduce the perceived discrepancy between subjective and objective sleep measures [47,48], which is a typical finding in insomnia sufferers [49].

As previous research has referred mainly to subjective results and there is a lack of randomized controlled trials (RCT) which investigate pre- to post changes in objective measures using internet-based interventions, we here aimed at assessing the efficacy of a minimally guided CBT-I-based online intervention compared to an active control group by implementing both subjective as well as objective sleep measures in our study.

## 2. Results

### 2.1. Subjective Sleep Parameters

#### 2.1.1. Insomnia Severity

**Figure 1:**
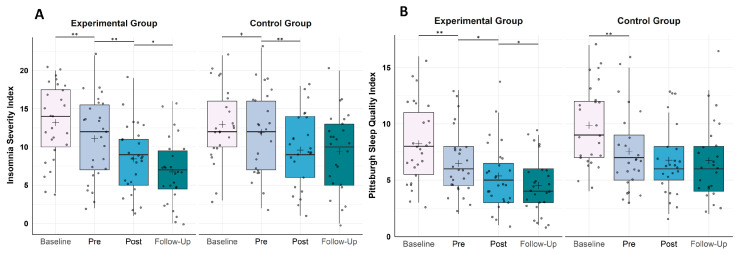
Boxplots showing the change in (**A**) insomnia symptoms assessed by the Insomnia Severity Index (ISI) and (**B**) subjective sleep quality assessed by the Pittsburgh Sleep Quality Index (PSQI). (**A**) In the experimental group (EG, *n* = 27), the severity of insomnia symptoms decreased significantly from baseline to pre-intervention as well as from pre-intervention to post-intervention (6 weeks later) and from post-intervention to follow-up (4 weeks later), whereas in the active control group (CG, *n* = 25), there was a trend of a decrease in the severity of insomnia symptoms from baseline to pre-intervention as well as a significant decrease from pre- to post-intervention. (**B**) In the experimental group, subjective sleep quality changed significantly from baseline to pre-intervention, from pre- to post-intervention and from post-intervention to follow-up, while in the active control group, subjective sleep quality decreased significantly from baseline to pre-intervention only. Higher values represent a stronger impairment in the respective measures. Horizontal lines represent the medians, and boxes represent the interquartile range, with whiskers depicting the 1.5 interquartile range. The black cross in the boxes corresponds to the mean. Baseline (T0), Pre-intervention (T1), Post-intervention (T2), Follow-up (T3). Asterisks indicate significance: ** *p* < 0.010, * *p* < 0.05, + *p* < 0.10.

A 4 × 2 repeated measures ANOVA with the within-subject factor TIME (T0, T1, T2, T3), the between-subject factor GROUP (EG, CG) and the dependent variable “insomnia severity” (assessed by the ISI) revealed a significant main effect of TIME (*F*(3, 150) = 34.33, *p* < 0.001, *η*^2^*p* = 0.407) and no significant main effect of GROUP (*F*(1, 50) = 0.89, *p* = 0.350, *η*^2^*p* = 0.017). There was a trend for a significant interaction effect of TIME x GROUP (*F*(3, 150) = 2.41, *p* = 0.069, *η*^2^*p* = 0.046). Subsequent post hoc Wilcoxon tests (cf. Figure 1A) revealed a significant decrease in insomnia severity in the EG from T0 to T1 (Baseline; *Z* = −3.24, *p* = 0.001, *r* = 0.62), from T1 to T2 (Intervention; *Z* = −3.02, *p* = 0.003, *r* = 0.58) as well as from T2 to T3 (Follow-up; *Z* = −2.11, *p* = 0.035, *r* = 0.41), while in the CG, there was a trend for a significant decrease in insomnia severity from T0 to T1 (Baseline; *Z* = −1.66, *p* = 0.096, *r* = 0.33) and a significant decrease from T1 to T2 (Intervention; *Z* = −2.65, *p* = 0.008, *r* = 0.53) but no change from T2 to T3 (Follow-up; *Z* = −0.04, *p* = 0.972).

#### 2.1.2. Subjective Sleep Quality

A 4 × 2 repeated measures ANOVA with the within-subject factor TIME (T0, T1, T2, T3), the between-subject factor GROUP (EG, CG) and the dependent variable “subjective sleep quality” (assessed by the PSQI) revealed a significant main effect of TIME (*F*(3, 150) = 33.51, *p* < 0.001, *η*^2^*p* = 0.401), a significant main effect of GROUP (*F*(1, 50) = 4.24, *p* = 0.045, *η*^2^*p* = 0.078) but no significant interaction effect of TIME × GROUP (*F*(3, 150) = 2.94, *p* = 0.486, *η*^2^*p* = 0.016). Subsequent post hoc Wilcoxon tests (cf. Figure 1B) revealed a significant increase in subjective sleep quality in the EG from T0 to T1 (Baseline; *Z* = −2.86, *p* = 0.004, *r* = 0.55), from T1 to T2 (Intervention; *Z* = −2.54, *p* = 0.011, *r* = 0.49) as well as from T2 to T3 (Follow-up; *Z* = −2.44, *p* = 0.015, *r* = 0.47), while in the CG, there was only a change from T0 to T1 (Baseline; *Z* = −3.01, *p* = 0.003, *r* = 0.60) but not from T1 to T2 (Intervention; *Z* = −1.64, *p* = 0.101) or T2 to T3 (Follow-up; *Z* = −0.19, *p* = 0.850).

### 2.2. Objective Sleep Parameters

#### 2.2.1. Wake after Sleep Onset

**Figure 2:**
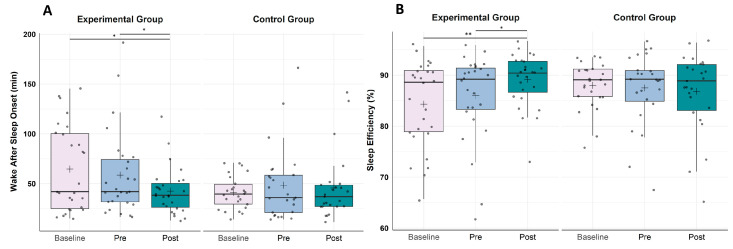
Boxplots showing the change in (**A**) objective wake after sleep onset (WASO) in minutes and (**B**) objective sleep efficiency (SE) in percentage assessed by ambulatory polysomnography. (**A**) In the experimental group (EG, *n* = 27), the time spent in wake after sleep onset decreased significantly from baseline to post-intervention as well as from pre- to post-intervention (6 weeks later). Higher values represent a longer wake after sleep onset duration. (**B**) In the experimental group, sleep efficiency increased significantly from baseline to post-intervention as well as from pre- to post-intervention (6 weeks later). Lower values represent worse sleep efficiency. Horizontal lines represent the medians, and boxes represent the interquartile range, with whiskers depicting the 1.5 interquartile range. The black cross in the boxes corresponds to the mean. Baseline (T0), Pre-intervention (T1), Post-intervention (T2). Control group (CG), *n* = 25. Asterisks indicate significance: ** *p* < 0.010, * *p* < 0.05.

A 3 × 2 repeated measures ANOVA with the within-subject factor TIME (T0, T1, T2), the between-subject factor GROUP (EG, CG) and the dependent variable ”wake after sleep onset” assessed by ambulatory PSG revealed no significant main effect of TIME (*F*(2, 100) = 1.96, *p* = 0.146, *η*^2^*p* = 0.038) or of GROUP (*F*(1, 50) = 1.92, *p* = 0.172, *η*^2^*p* = 0.037) but a significant interaction effect of TIME × GROUP (*F*(2, 100) = 3.12, *p* = 0.048, *η*^2^*p* = 0.059). Subsequent post hoc *t*-tests revealed (cf. Figure 2A) no significant change in wake after sleep onset in the EG from T0 to T1 (Baseline; *t*(26) = 0.77, *p* = 0.447) but from T1 to T2 (Intervention; *t*(26) = 2.10, *p* = 0.046, *r* = 0.38) as well as from T0 to T2 (*t*(26) = 2.66, *p* = 0.013, *r* = 0.46), while in the CG, no change was found in wake after sleep onset, neither from T0 to T1 (Baseline; *t*(24) = −0.95, *p* = 0.354) nor from T1 to T2 (Intervention; *t*(24) = 0.41, *p* = 0.683) or T0 to T2 (*t*(25) = −0.41, *p* = 0.689).

#### 2.2.2. Sleep Efficiency

A 3 × 2 repeated measures ANOVA with the within-subject factor TIME (T0, T1, T2), the between-subject factor GROUP (EG, CG) and the dependent variable “sleep efficiency” revealed no significant main effect of TIME (*F*(2, 100) = 1.56, *p* = 0.214, *η*^2^*p* = 0.030) or of GROUP (*F*(1, 50) = 0.37, *p* = 0.544, *η*^2^*p* = 0.007) but a significant interaction effect of TIME x GROUP (*F*(2, 100) = 4.13, *p* = 0.019, *η*^2^*p* = 0.076). Subsequent post hoc *t*-tests (cf. Figure 2B) revealed no significant change in sleep efficiency in the EG from T0 to T1 (Baseline; *t*(26) = −1.06, *p* = 0.298) but from T1 to T2 (Intervention; *t*(26) = −2.07, *p* = 0.049, *r* = 0.38) as well as from T0 to T2 (*t*(26) = −2.92, *p* = 0.007, *r* = 0.50), while in the CG, there was no change in sleep efficiency, neither from T0 to T1 (Baseline; *t*(24) = 0.34, *p* = 0.739) nor from T1 to T2 (Intervention; *t*(24) = 0.57, *p* = 0.574) or T0 to T2 (*t*(25) = 0.55, *p* = 0.590).

#### 2.2.3. Total Sleep Time

A 3 × 2 repeated measures ANOVA with the within-subject factor TIME (T0, T1, T2), the between-subject factor GROUP (EG, CG) and the dependent variable “total sleep time” assessed by ambulatory PSG revealed no significant main effect of TIME (*F*(2, 100) = 1.23, *p* = 0.296, *η*^2^*p* = 0.024) or of GROUP (*F*(1, 50) = 0.16, *p* = 0.694, *η*^2^*p* = 0.003) as well as no significant interaction effect of TIME x GROUP (*F*(2, 100) = 0.02, *p* = 0.984, *η*^2^*p* = 0.000).

## 3. Discussion

The aim of this RCT was to investigate subjective as well as objective changes of sleep after implementation of (i) a minimally guided 6-week online program based on the principles of CBT-I in an experimental group in comparison to (ii) an active control group receiving no therapy-specific content but sleep hygiene-promoting material as well as instructions for healthy daily movement and daylight exposure.

### 3.1. Changes in Subjective Measures of Sleep (Questionnaires)

In the EG we found significant (and in the CG tendential) changes regarding the severity of insomnia symptoms assessed via ISI during the 2-week baseline, although participants did not know which kind of group they belonged to until T1 (pre-intervention). Participants did, however, fill in the sleep diary during the baseline period, and changes in the subjective perspective of their own sleep can therefore partly be explained through their more pronounced confrontation with their own sleep [50]. Furthermore, literature has shown that even mere enrolment in a study protocol can have an impact on the subjective feeling of the participants due to the expectation of being under professional guidance (Hawthorne effect). As expected, we found further improvements in insomnia symptom severity during the intervention period in the EG. Interestingly, the CG also showed a substantial decrease in insomnia symptoms during the intervention period, leading to the conclusion that “minimal interventions” such as sleep hygiene instructions can also lead to a subjective symptom reduction and consequently to sleep improvements, e.g., [51,52,53]. In the EG, however, there was a further improvement during the follow-up period (from *M*_T2_ = 8.44 to *M*_T3_ = 6.74; *p* = 0.035), which was not observed in the active CG. Furthermore, in the EG alone, there was a change of 7 points (regarding the *Mdn*) from T0 to T3, which exceeds the recommended 6-point median change needed to assume a clinically relevant improvement [54]. On average, in the EG, we found an improvement from *M*_T0_ = 13.19 points to *M*_T3_ = 6.74 points, meaning that participants in the EG improved from the category of “subthreshold insomnia” to a clinically not meaningful value, while in the CG, participants remained in the category of “subthreshold insomnia” until the follow-up assessment (*M*_T3_ = 9.44). In the EG, 85.2% started (i.e., T0) with a clinically meaningful ISI-value > 7, which decreased to 63% after intervention (i.e., T2) and to 29.6% at follow-up (i.e., T3).

Subjective sleep quality according to PSQI showed a similar pattern. In the EG, we found improvements from pre- to post-intervention as well as from post-intervention to follow-up, while in the CG, we did not observe such changes. On average, the EG improved from *M*_T0_ = 8.22 points to *M*_T3_ = 4.52 points, meaning that participants in the EG switched from the category of “poor sleep quality” to a clinically not-meaningful value (PSQI ≤ 5), while in the CG, the group mean remained in the category of “poor sleep quality” until the follow-up assessment (*M*_T3_ = 6.72). In the EG, 74.1% started (i.e., T0) with a clinically meaningful PSQI-value > 5, which decreased to 40.7% after intervention (i.e., T2) and to 29.6% at follow-up (i.e., T3). Our results on subjective measures confirm the findings of previous studies showing improved insomnia severity and subjective sleep quality in participants suffering from impaired sleep after using digital CBT-I based interventions [39,55].

### 3.2. Changes in Objective Measures of Sleep (PSG)

What many comparable previous studies lack is the additional and systematic consideration of objectively (PSG-) derived measures. Using one night of ambulatory PSG at each main measurement time point (T0, T1, T2), we investigated the changes in objective sleep parameters over the study period and found significant improvements in medium effect size for WASO (*r* = 0.38) and SE (*r* = 0.38) from pre- to post-intervention in the EG, while in the CG, no changes were observed. Using an internet-based intervention with only minimal guidance, our findings are in accordance with the work of Krystal and Edinger [43] who found improvements in the same parameters after the administration of onsite CBT-I. Furthermore, our results correspond to the outcome of a meta-analysis from Okajima and colleagues [28], who also found medium effect sizes for an improvement in objective WASO (*d* = 0.42) and SE (*d* = 0.53) derived from PSG and/or actigraphy at the end of F2F CBT-I.

In our sample, WASO reduced on average from *M*_T0_ = 64.6 min to *M*_T2_ = 42.5 in participants of the EG, which, according to the National Sleep Foundation, would, however, not qualify as a clinically relevant outcome, as a maximum wake after sleep onset from 20 min is set as a criterion [56]. Nevertheless, a reduction in WASO of 34% can be considered as a relevant improvement regarding the individual’s quality of life. Furthermore, SE increased in the EG from *M*_T0_ = 84.32% to *M*_T2_ = 89.18%, which can be classified as an appropriate indicator of good sleep quality according to the National Sleep Foundation (SE ≥ 85%) [56]. Detecting changes in objective sleep measures after the administration of an iCBT-I-based program is notable, as many studies have not found improvements in PSG-derived measures regardless of the CBT-I setting and especially not in the iCBT-I settings, e.g., [40]. In our opinion, however, objective changes are desirable and relevant as only in these cases can one assume that the respective intervention also alleviates the adverse consequences of non-restorative sleep on a physiological level. To our knowledge, the only existing study examining the effects of a digital CBT-I-based program on objective sleep measures was conducted by Reilly et al. [57]. Having measured sleep objectively by using a home-sleep apnea device, which also assessed basic sleep parameters, they found similarly significant (though small) changes in sleep efficiency after the application of a 6-week app program in a sample of veterans suffering from chronic insomnia. However, further objective parameters were not examined in this trial.

As, apart from our work, there is very little research on the effects of internet-based or digital CBT-I which includes objective outcome measures, further research is urgently needed.

### 3.3. Limitations and Future Directions

This study has some limitations which need mentioning. As most of the study was carried out during the COVID-19 pandemic, participants were confronted with various additional stressors (e.g., home office, lockdowns, confinements), which were out of our control. The inability to influence these environmental factors may have led to enhanced anxiety and rumination, which has been shown to lead to worse sleep quality [58,59]. Therefore, it is possible that participants’ sleep during the study period was additionally impaired by these circumstances and that, as a result, the efficacy of the program may have been altered. As the pandemic situation also repeatedly kept us from continuing data acquisition (as in-laboratory investigations were prohibited in times of high incidences), the present dataset could not be collected within a continuous time frame; hence, data collection had to be spread over the study period. This may have led to unspecific time effects, which we were beyond our control.

Furthermore, it must be mentioned that the sample investigated in this study was not a primary clinical sample; therefore, the improvement in various parameters may have been smaller than would have been the case in a fully clinically impaired sample. Nevertheless, it is striking that even this general community sample still showed increased values of PSQI (>5, 83.0%) as well as of ISI (>7, 88.7%). This again illustrates the fact that a large proportion of the general population is obviously suffering from sleep problems without having sufficient access to effective treatment. For our study, we chose rather liberal inclusion criteria and, therefore, had an ecologically valid but not very homogenous sample.

As is typical for digital programs, which are often without guidance, it is difficult to control the administration and intensity with which the exercises and tasks are completed. The analyses reveal that participants spent on average 97 min on one module and that participants of the EG carried out on average five relaxation exercises per module. One of the main tasks of the active CG was to spend more time in daylight and to increase movement and exercises. In the present study the only possibility to track whether these requirements were met was by monitoring the daily step count from the activity tracker. What we could not control was light exposure and in which way the EG executed the CBT-I-based exercises.

A further point to be mentioned is that the objectively derived measures were based only on the recording of one single night of ambulatory PSG during each of the three measurement time points. Therefore, possible first-night effects and distortions may have been present; however, we consider the baseline night to also be the adaptation night, so that from pre- to post-intervention, clean and natural objective sleep measures can be expected.

## 4. Materials and Methods

### 4.1. Participants

A total of 53 volunteers between 21 and 71 years (*M* = 44.6 ± 12.5), 27 female, were randomly assigned to receive either a minimally guided, 6-week web-based program established on CBT-I components (https://www.gesunderschlaf.coach (accessed on 10 September 2023); EG, *n* = 27) or to take part in an active control group (CG, *n* = 26). An online automated system was used for random group allocation. Participants were 18 years or older, reported impaired sleep quality and had no acute psychiatric or neurological disorder. Descriptive statistics are reported in Supplementary Material, Table S1. Most of the participants (83%, *n* = 44) reported a clinically relevant value > 5 on the Pittsburgh Sleep Quality Index (PSQI) [60] at study entrance (i.e., T0), which points at poor sleep quality, while even 40.9% (*n* = 18) of those had a value > 10, indicating chronic sleep disturbances according to the PSQI. Regarding the Insomnia Severity Index (ISI) [61], 88.7% (*n* = 47) had a clinically relevant value > 7, suggesting the presence of “subthreshold insomnia”. Nearly half (46.8%, *n* = 22) fell into the category of “moderate clinical insomnia” according to ISI. For a detailed overview of the number and percentage of participants exceeding the relevant cut-off scores of PSQI and ISI categories per measurement time point (T0–T3), refer to Supplementary Material, Table S2. Recruitment was performed via social media and a local Austrian radio proclamation. Data collection was carried out between March 2021 and April 2022. The study was approved by the ethical committee of the University of Salzburg (GZ 46/2020) and was conducted in accordance with the Declaration of Helsinki. All participants gave informed consent prior to enrolment in the study protocol.

### 4.2. Study Design and Procedure

**Figure 3:**
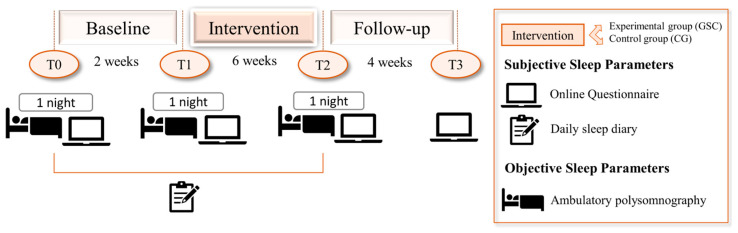
Study design. Participants’ sleep was assessed subjectively via questionnaires at baseline (T0), pre-intervention (T1), post-intervention (T2) as well as follow-up (T3). Furthermore, sleep was assessed objectively via ambulatory polysomnography at baseline (T0) as well as at pre- and post-intervention (T1–T2). A sleep diary was completed continuously during both the baseline and intervention period (T0–T2).

Participants’ current (subjective as well as objective) sleep state was assessed at “baseline” (cf. Figure 3; T0) prior to the start of the intervention. For a subjective assessment of the status quo, an online entrance questionnaire was used, which included demographic data, questions regarding medical history, the PSQI, the ISI, the Dysfunctional Beliefs and Attitudes about Sleep scale (DBAS-16) [62] as well as the brief version of the WHO’s Quality-of-Life Questionnaire (WHOQOL-Bref) [63]. Data of the DBAS-16 and the WHOQOL-Bref, however, were not analyzed as part of this publication. Objective sleep parameters were obtained at baseline from one night of ambulatory polysomnography (PSG). The two weeks between baseline and pre-intervention assessment served as the control condition, which allowed for the observation of possible changes due to treatment-unspecific spontaneous remission. Just before the beginning of the intervention, the severity of insomnia symptoms and subjective sleep quality (measured with the ISI and PSQI, respectively) as well as another PSG night were assessed again (Pre-Intervention; T1). Finally, to determine the efficacy of the GSC program, objective and subjective values were measured at the end of the intervention period of six weeks (Post-Intervention; T2). For subjective data, a follow-up assessment one month after post-intervention (Follow-up; T3) was added. During study participation, i.e., the baseline phase as well as the intervention phase, participants completed an online sleep diary every morning to continuously assess (subjective) sleep parameters. During the whole study period (T0–T2), participants wore an activity tracker (Mi Band 3, Xiaomi Tech, Peking, China) to record their daily step count.

#### 4.2.1. Intervention Program “GesunderSchlaf.Coach”

The web-based program “GesunderSchlaf.Coach” is based on the core principles of CBT-I and was available in German during the study period over an interactive website. The program is based on the therapy manual by Scharfenstein and Basler [64] and consists of six modules, which include components of psychoeducation, relaxation techniques, cognitive restructuring, sleep hygiene and stimulus control. The participants worked independently on one module per week, spending about 10–15 min per day on the content of the respective module. This low-threshold form of delivery enables the integration of the exercises into everyday life. Each module can be divided into four categories. First, different relaxation exercises are presented, then psychoeducational information is provided, after which advice for better sleep is listed and, finally, sleep promoting interventions are suggested. Once a week, after completing the corresponding module, participants were invited to contact the study team over the website (via chat) for 30 min to clarify open questions and to promote reflection on what had been learned.

#### 4.2.2. Active Control Group

The online program provided to the CG does not include cognitive-behavioral elements, but incorporates approaches that have been shown to be sleep-promoting in the past, namely (i) exposure to daylight (at least 30 min per day), (ii) moderate physical activity (at least 6000 steps per day) and (iii) psychoeducation and sleep hygiene rules. Similar to the GSC group, the program was organized in six modules, and participants were instructed to spend about 10–15 min per day on the current module. Physical activity was measured through step count, which was assessed by the use of the Xiaomi Mi Band 3. Participants of the CG received the same weekly chat offer as the EG. About every second participant of the entire sample (i.e., EG and CG) made use of that offer on a regular weekly basis. For ethical reasons, the CG also received full access to the GSC program after the end of the study period.

### 4.3. Materials and Measures

#### 4.3.1. Subjective Sleep Assessment

##### Insomnia Severity

For measuring the severity of insomnia symptoms, the Insomnia Severity Index (ISI) [61,65] was used and assessed at four different time points (T0–T3). It consists of seven items, which are answered on a 5-point Likert scale. A total score ranging from 0 to 28 provides information about the degree of impairment caused by insomnia symptoms. A score from 0 to 7 is considered as normal/clinically not relevant, 8 to 14 as sub-threshold insomnia, 15 to 21 as moderate insomnia and above 21 as severe clinical insomnia.

##### Sleep Quality

The Pittsburgh Sleep Quality Index (PSQI) [66] was used to measure subjective sleep quality at four different time points (T0–T3). It consists of 18 items which result in a global score lying between 0 and 21. A cut-off score of 5 divides between good and bad sleepers, with a score > 5 being considered as “poor sleep quality”; a score > 10 is rated as “chronic sleep disorder”.

##### Sleep Diary

For continuously assessing changes in subjective sleep quality, a daily sleep diary was obtained online. The main parameters assessed here were Sleep Onset Latency (SOL), Wake After Sleep Onset (WASO), Total Sleep Time (TST) and Total Bedtime (TBT). Sleep diary data were not analyzed as part of this publication.

#### 4.3.2. Objective Sleep Assessment

##### Ambulatory Polysomnography

Sleep recordings were obtained using an ambulatory amplifier system (Alphatrace, Becker Meditec; Karlsruhe, Germany) with a sampling rate of 512 Hz. The electroencephalographic (EEG) signal was obtained by attaching gold-plated scalp electrodes (Grass Technologies, Astro-Med GmbH, Rodgau, Germany) according to the international 10–20 system on channels F3, Fz, F4, C3, C4, P3, Pz, P4, O1 and O2 and referenced online against Cz. To assess electrooculography, four electrodes were mounted: two horizontals placed above the right and left outer canthi, and two verticals placed above and below the right eye. The electromyography channel was derived from two electrodes over the left and right musculus mentalis. Additionally, two more electrodes were placed below the right clavicle and on the left side below the pectoral muscle at the lower edge of the left rib cage for measuring the electrocardiographic signal. The (ambulatory) EEG montage was performed at the sleep laboratory of the University of Salzburg; after the montage, participants were able to sleep with the system in their home environment. After returning the equipment, the recordings were down-sampled to 128 Hz using BrainVision Analyzer (Brain Products GmbH, Gilching, Germany), re-referenced offline to averaged channels A1 and A2 at the mastoids as well as pre-filtered according to the American Academy of Sleep Medicine (AASM) filter settings for routine PSG recordings [67]. Sleep was automatically scored in 30 s epochs using standard AASM scoring criteria (Sleepware G3, Koniklijke Philips N.V.; Eindhoven, The Netherlands). The G3 software is considered to be non-inferior to manual human staging and can be readily used without the need for manual adjustments [68]. The results were used to generate the traditional PSG parameters (TST, WASO, SOL, SE) according to the AASM, with WASO being defined as the duration from the first sleep epoch to the time out of bed, SOL being defined as the duration from “lights off” to the first (N1) sleep epoch and sleep efficiency being defined as TST/TIB ∗ 100.

### 4.4. Statistical Analyses

Statistical analyses were performed using SPSS 29 (SPSS Inc., Chicago, IL, USA) and R.Studio, Version 2023.03.0 (RStudio PBC, Boston, MA, USA). Analyses were mainly based on mixed analyses of variance (ANOVA). The significance level was set to *p* < 0.05. As suggested by Wasserstein et al. [69], we interpreted the overall pattern rather than focusing on individual *p*-values. Therefore, we also interpreted *p*-values of 0.05 < *p* ≤ 0.10 as statistical trends if they were in line with the overall pattern. For the measure of effect size, either partial eta squared (*η*^2^*p*) or the correlation coefficient (*r*) were provided, depending on the inferential statistics applied. For post hoc comparisons, parametric tests (*t*-tests) or Wilcoxon rank-sum tests were applied (depending on the distribution of the data). Two-tailed critical *p*-values are reported. Descriptive statistics on the outcome variables can be found in the Supplementary Material (subjective sleep parameters: Table S3; objective sleep parameters: Table S4). There was no significant difference in the investigated outcome variables between the groups at pre-intervention (cf. Supplementary Material, Table S5).

## 5. Conclusions

This randomized controlled trial found significant improvements in subjective as well as objective sleep measures in an experimental group after the administration of a 6-week internet-based CBT-I program, while an active control group with only sleep hygiene instructions showed improvements in the severity of insomnia symptoms and non-intervention-specific effects in subjective sleep quality. Therefore, even online-administered minimal interventions building on sleep-promoting habits (exercise, daylight, sleep hygiene) seem to have a subtle positive effect on subjective sleep perception and symptoms of insomnia. Furthermore, changes in objective sleep parameters, which are necessary for associated physiological benefits, seem to be possible only after a full-term CBT-I-based intervention. Here, digital alternatives such as iCBT-I also seem to help.

In summary, the improvement in both subjective and objective measures we found in the EG is notable, especially as previous studies (i) were not able to detect any changes in objective sleep data or (ii) did not involve objective (PSG) measures at all cf., [70]. We provide initial evidence for the potential benefits of iCBT-I interventions on both subjective and objective measures of sleep, which is essential for drawing conclusions about treatment efficacy on a physiological level. Furthermore, our findings support the beneficial effect of a minimally guided, internet-based intervention for improving sleep over the course of 6 weeks. Digital programs should be used to complement classical F2F CBT-I at an early stage in order to prevent the chronification of sleep problems, which subsequently may close the current treatment gap.

## Data Availability

The datasets generated during and/or analyzed during the current study are available from the corresponding author on reasonable request.

## Supplementary Materials

The following supporting information can be downloaded at: https://www.mdpi.com/article/10.3390/clockssleep5040039/s1, Table S1: Demographic characteristics of participants; Table S2: Overview of the number (*n*) and percentage (%) of participants exceeding the cut-off scores of the Pittsburgh Sleep Quality Index (PSQI; >5 poor sleep quality, >10 chronic sleep disorder) and the Insomnia Severity Index (ISI; >7 subthreshold insomnia, >14 moderate severe clinical insomnia) for all measurement time points (T0–T3); Table S3: Mean (*M*), median (*Mdn*) and standard deviation (*SD*) for all measurement time points (T0–T3) regarding the (subjective) outcome measures of insomnia symptom severity assessed using the Insomnia Severity Index (ISI) and subjective sleep quality assessed using the Pittsburgh Sleep Quality Index (PSQI) in the experimental group (EG) in comparison to the control group (CG); Table S4: Mean (*M*), median (*Mdn*) and standard deviation (*SD*) for all measurement time points (T0–T3) regarding the objective outcome measures of wake after sleep onset (minutes) and sleep efficiency (%) assessed using ambulatory polysomnography in the experimental group (EG) in comparison to the control group (CG); Table S5: Differences between the groups in all outcome variables at measurement time point T1 (Pre-Intervention).
