# Supplementary material for: On the Efficacy of a CBT-I-Based Online Program for Sleep Problems: A Randomized Controlled Trial
Source: Clocks Sleep. 2023 Oct 9;5(4):590–603. doi: 10.3390/clockssleep5040039 (PMC10594462; doi:10.3390/clockssleep5040039)
Supplement: Supplementary file 1 [file clockssleep-05-00039-s001.zip › clockssleep-2613649-supplementary.pdf]

SUPPLEMENTARY MATERIAL

On the efficacy of a CBT-I-based online program for sleep problems: a randomized controlled trial

Esther-Sevil Eigl[1], Theresa Hauser[1], Pavlos I. Topalidis[1] & Manuel Schabus[1,2]

[1] Laboratory for Sleep, Cognition & Consciousness Research, Paris-Lodron University of Salzburg, Austria
[2] Centre for Cognitive Neuroscience Salzburg (CCNS), Paris-Lodron University of Salzburg, Austria

Corresponding Author:

Manuel Schabus,
Hellbrunnerstr. 34
A-5020 Salzburg, Austria
manuel.schabus@plus.ac.at

Table S1. Demographic characteristics of participants.

**Response Option**	**Total**		**EG**		**CG**	
	n	%	*n*	%	*n*	%
**Sex**						
Male	26	49.1	13	48.1	13	50.0
Female	27	50.9	14	51.9	13	50.0
**Educational level**						
University degree	26	49.1	9	33.3	17	65.4
High school	10	18.9	7	25.9	3	11.5.
Secondary school	2	3.8	2	7.4		
Educational training	6	11.3	3	11.1	3	11.5
Vocational high school	7	13.2	6	22.2	1	3.8
Higher general-education school	2	3.8			2	7.7
**Marital status**						
Married	28	52.8	14	51.9	14	53.8
Partnership	11	20.8	6	22.2	5	19.2
Single	10	18.9	5	18.5	5	19.2
Divorced	3	1.9	2	7.4	1	3.8
Widowed	1	1.9			1	3.8

Note. N_{Total} = 53, Experimental Group = EG; *n* = 27, Control Group = CG; *n* = 26; *grey boxes:* response option was not chosen in this group.

Table S2. Overview of the number (*n*) and percentage (%) of participants exceeding the cut-off-scores of the Pittsburgh Sleep Quality Index (PSQI; >5 poor sleep quality, >10 chronic sleep disorder) and the Insomnia Severity Index (ISI; >7 subthreshold insomnia, >14 moderate severe clinical insomnia) for all measurement time points (T0-T3).

**Outcome variable**	**Total**				**EG**				**CG**			
**PSQI**	**>5**		**>10**		**>5**		**>10**		**>5**		**>10**	
	n	**%**	***n***	**%**	***n***	**%**	***n***	**%**	***n***	**%**	***n***	**%**
T0	44	83	18	34	46	74.1	7	25.9	24	92.3	11	42.3
T1	34	64.2	8	15.1	15	55.6	3	11.1	19	73.1	5	19.2
T2	29	54.7	6	11.3	11	40.7	2	7.4	18	69.2	4	15.4
T3	*23*	*44.2*	*3*	*5.8*	8	29.6			*15*	*60.0*	*3*	*12*
**ISI**	**>7**		**>14**		**>7**		**>14**		**>7**		**>14**	
	n	**%**	***n***	**%**	***n***	**%**	***n***	**%**	***n***	**%**	**n**	**%**
T0	47	88.7	22	41.5	23	85.2	12	44.4	24	92.3	10	38.5
T1	36	67.9	18	34	18	66.7	8	29.6	18	69.2	10	38.5
T2	36	67.9	6	11.3	17	63	2	7.4	19	73.1	4	15.4
T3	*24*	*46.2*	*5*	*9.6*	8	29.6	2	7.4	*16*	*64*	*3*	*12*

Note. N_{Total} = 53, Experimental Group = EG; *n* = 27, Control Group = CG; *n* = 26. *Italic values* correspond to the respective n_{Group}= -1, as of a missing value at T3 (i.e., N_{Total} = 52, n_{CG} = 25). *Grey boxes:* response option was not chosen in this group.

Table S3. Mean (*M*), median (*Mdn*) and standard deviation (*SD*) for all measurement time points (T0-T3) regarding the (subjective) outcome measures of insomnia symptom severity assessed using the Insomnia Severity Index (ISI) and subjective sleep quality assessed using the Pittsburgh Sleep Quality Index (PSQI) in the experimental group (EG) in comparison to the control group (CG).

	**EG**			**CG**		
**Outcome variable**	*M*	*Mdn*	*SD*	*M*	*Mdn*	*SD*
ISI T0	13.19	14.00	4.93	12.96	12.00	4.88
ISI T1	11.07	12.00	5.37	11.92	12.00	5.50

ISI T2	8.44	9.00	4.47	9.60	9.00	4.77
ISI T3	6.74	7.00	4.26	9.44	10.00	5.06
PSQI T0	8.22	8.00	3.51	9.88	9.00	3.64
PSQI T1	6.48	6.00	2.94	7.56	7.00	3.54
PSQI T2	5.37	5.00	2.98	6.76	6.00	3.11
PSQI T3	4.52	4.00	2.49	6.72	6.00	3.43

Note. Experimental Group = EG; *n* = 27, Control Group = CG; *n* = 25. Baseline (T0), Pre-intervention (T1), Post-intervention (T2), Follow-up (T3).

Table S4. Mean (*M*), median (*Mdn*) and standard deviation (*SD*) for all measurement time points (T0-T3) regarding the objective outcome measures of wake after sleep onset (minutes) and sleep efficiency (%) assessed using ambulatory polysomnography in the experimental group (EG) in comparison to the control group (CG).

		**EG**			**CG**	
**Outcome variable**	*M*	*Mdn*	*SD*	*M*	*Mdn*	*SD*
WASO T0	64.60	42.00	43.18	41.22	39.50	16.54
WASO T1	58.59	42.00	42.98	48.41	36.00	37.03
WASO T2	42.50	38.30	23.47	45.72	37.00	33.12
SE T0	84.32	88.60	8.46	88.00	89.10	4.53
SE T1	86.00	89.20	8.61	87.50	89.20	7.10
SE T2	89.18	90.40	5.17	86.82	88.90	7.65

Note. Experimental Group = EG; *n* = 27, Control Group = CG; Baseline (T0), Pre-intervention (T1), Post-intervention (T2), Follow-up (T3).

Table S5. Differences between the groups in all outcome variables at measurement time point T1 (Pre-Intervention).

	EG			CG				
**Outcome variable (T1)**	*M*	*Mdn*	*SD*	*M*	*Mdn*	*SD*	*Z-/T-value*	*p-value*
ISI	11.07	12.00	5.37	11.92	12.00	5.50	-*.560*	.575
PSQI	6.48	6.00	2.94	7.56	7.00	3.54	*-1.078*	.281
WASO	58.59	42.00	42.98	48.41	36.00	37.03	.911	.367
SE	86.00	89.20	8.61	87.50	89.20	7.10	-.684	.497

Note. Mean (*M*), median (*Mdn*), standard deviation (*SD*). ISI = Insomnia Severity Index, PSQI = Pittsburgh Sleep Quality Index, WASO = Wake After Sleep Onset, SE = Sleep efficiency. T1 = Pre-Intervention. Experimental Group = EG; *n* = 27, Control Group = CG; *n* = 25. *Italic values* correspond to non-parametric Z-values.
